# Contactin‐6‐deficient male mice exhibit the abnormal function of the accessory olfactory system and impaired reproductive behavior

**DOI:** 10.1002/brb3.2893

**Published:** 2023-03-01

**Authors:** Wei Zhang, Huiling Huang, Ailing Gui, Di Mu, Tian Zhao, Hongtao Li, Kazutada Watanabe, Zhicheng Xiao, Haihong Ye, Yiliang Xu

**Affiliations:** ^1^ Department of Medical Genetics and Developmental Biology, School of Basic Medical Sciences, Beijing Key Laboratory of Neural Regeneration and Repair Capital Medical University Beijing China; ^2^ State Key Laboratory of Membrane Biology, College of Life Sciences Peking University Beijing China; ^3^ Department of Bioengineering Nagaoka University of Technology Nagaoka Niigata Japan; ^4^ The Key Laboratory of Stem Cell and Regenerative Medicine, Institute of Molecular and Clinical Medicine Kunming Medical University Kunming China; ^5^ Department of Anatomy and Developmental Biology Monash University Clayton Melbourne Australia

**Keywords:** accessory olfactory system, contactin‐6, dendrodendritic synapse, reproductive behavior

## Abstract

**Introduction:**

Contactin‐6 (CNTN6), also known as NB‐3, is a neural recognition molecule and a member of the contactin subgroup of the immunoglobulin superfamily. Gene encoding CNTN6 is expressed in many regions of the neural system, including the accessory olfactory bulb (AOB) in mice. We aim to determine the effect of CNTN6 deficiency on the function of the accessory olfactory system (AOS).

**Methods:**

We examined the effect of CNTN6 deficiency on the reproductive behavior of male mice through behavioral experiments such as urine sniffing and mate preference tests. Staining and electron microscopy were used to observe the gross structure and the circuitry activity of the AOS.

**Results:**

*Cntn6* is highly expressed in the vomeronasal organ (VNO) and the AOB, and sparsely expressed in the medial amygdala (MeA) and the medial preoptic area (MPOA), which receive direct and/or indirect projections from the AOB. Behavioral tests to examine reproductive function in mice, which is mostly controlled by the AOS, revealed that *Cntn6* ^−/−^ adult male mice showed less interest and reduced mating attempts toward estrous female mice in comparison with their *Cntn6* ^+/+^ littermates. Although *Cntn6* ^−/−^ adult male mice displayed no obvious changes in the gross structure of the VNO or AOB, we observed the increased activation of granule cells in the AOB and the lower activation of neurons in the MeA and the MPOA as compared with *Cntn6* ^+/+^ adult male mice. Moreover, there were an increased number of synapses between mitral cells and granule cells in the AOB of *Cntn6* ^−/−^ adult male mice as compared with wild‐type controls.

**Conclusion:**

These results indicate that CNTN6 deficiency affects the reproductive behavior of male mice, suggesting that CNTN6 participated in normal function of the AOS and its ablation was involved in synapse formation between mitral and granule cells in the AOB, rather than affecting the gross structure of the AOS.

## Graphical abstract

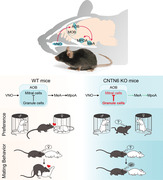

## 1 INTRODUCTION

Immunoglobulin cell adhesion molecules (IgCAMs), mainly expressed in the neural system, play important roles in the development of the neural system, which demands highly elaborate cooperation among different cell types and molecules. Interaction of neuronal IgCAMs among themselves by homophilic or heterophilic binding has been shown to be involved in neurite outgrowth, axon guidance, neuronal migration, synaptic plasticity, and memory (Dalva et al., 2007; Doherty et al., 1995; Maness et al., 2007; Murase et al., 1999; Rubenstein, 2011).

Contactin‐6 (CNTN6), also known as NB‐3, is a neuronal IgCAMs that promotes neurite outgrowth and synaptogenesis. It contains six immunoglobulin‐like domains followed by four fibronectin type III domains and is attached to the cell membrane by a glycosylphosphatidylinositol anchor. *Cntn6* is mainly expressed in the neural system, including the accessory olfactory bulb (AOB), cerebral cortex, thalamus, piriform cortex, hippocampus, and cerebellum in mice (Lee et al., 2000; Takeda et al., 2003). CNTN6 regulates the orientation of apical dendrites in layer V pyramidal neurons (Ye et al., 2008) and formation of the corticospinal tract in mice (Huang et al., 2011, 2012; Mercati et al., 2013). CNTN6 is essential for glutamatergic synapse formation in the hippocampus and cerebellum (Sakurai et al., 2010) and also facilitates the production of oligodendrocytes by progenitor cells (Cui et al., 2004; Hu et al., 2006; Wong et al., 2014). Although there is a robust expression of *Cntn6* in the AOB of mice (Lee et al., 2000; Takeda et al., 2003), its involvement in the development and function of the accessory olfactory system (AOS) remains unexplored.

Mice have two parallel but partially overlapping olfactory systems, the main olfactory system (MOS) and the AOS. Traditionally, the MOS is thought to detect a broad spectrum of chemicals, whereas the AOS is suitable for the detection of pheromones emitted by animals, such as those in urine, tears, and saliva (Firestein, 2001; Keverne, 1999) or semiochemicals (Halpern et al., 2003; Isogai et al., 2011; Kirschenbaum et al., 1986; Riviere et al., 2009). However, there is no strict division, as some stimuli are detected by both olfactory systems. Recent studies also reveal a combined, synergic interaction between the MOS and the AOS (Matsuo et al., 2015; Mucignat‐Caretta et al., 2012; Vargas‐Barroso et al., 2017; Xu et al., 2005). For a typical AOS, the vomeronasal organs (VNOs) have two layers that contain four populations of vomeronasal sensory neurons (VSNs). VSNs located in the apical layer express vomeronasal receptor 1 (V1Rs), which signal through the Gαi2 subunit of heterotrimeric G proteins (Chamero et al., 2012; Dulac et al., 1995; Jia & Halpern, 1996; Montani et al., 2013), some members of the formyl peptide receptor family (Riviere et al., 2009) and canonical odorants receptors (Levai et al., 2006). In contrast, VSNs located in the basal layer express vomeronasal receptor 2 (V2Rs), which signal through the Gαo subunit (Chamero et al., 2011, 2012; Herrada et al., 1997; Jia & Halpern, 1996; Montani et al., 2013). This dichotomy of the VNO is maintained along the axonal projections of VSNs to their target, the AOB. Apical VSNs innervate the anterior glomeruli of the AOB, whereas basal VSNs project their axons to the posterior glomeruli of the AOB (Halpern et al., 1998, 1995; Jia & Halpern, 1996). Mitral cells in the AOB receive pheromone stimuli collected by the VNO, and then project their axons to the medial amygdala (MeA), which then relays information to the medial preoptic area (MPOA) to regulate animal behavior, including sexual behavior and social dominance (Dulac et al., 2006; Martinez et al., 2013; Zufall et al., 2007). Besides MeA, bed nuclei of the stria terminalis (BNST), specifically the posterior part (BNSTp) and cortical amygdala, are also the projection target of mitral cells (Gutierrez‐Castellanos et al., 2014; Mohedano‐Moriano et al., 2007). The inhibitory feedback, formed by the microcircuits of the reciprocal dendrodendritic synapses between mitral and granule cells in the AOB, regulates the activity of mitral cells (Kaba & Keverne, 1988; Li et al., 1990).

Previous studies have demonstrated that *Cntn6* is expressed in the AOB (Lee et al., 2000). To assess the potential role of CNTN6 in the AOS, we used *Cntn6*‐deficient mice in which exon 2 of the *Cntn6* gene is replaced by *LacZ* and *Neo*, and these mutant mice produce β‐galactosidase instead of CNTN6 (Takeda et al., 2003). Here, we found that *Cntn6* is highly expressed in the VNO and the AOB. *Cntn6* ^−/−^ adult male mice showed less interest and reduced mating behavior toward estrous female mice as compared with their *Cntn6* ^+/+^ littermates. *Cntn6* ^−/−^ adult male mice showed no obvious changes in the gross structure of the VNO or the AOB. However, we observed a lower activation of the MeA and the MPOA in *Cntn6* ^−/−^ adult male mice, which is likely due to an increased number of excitatory synapses between mitral and granule cells, and subsequent more activated granule cells in the AOB as compared with *Cntn6* ^+/+^ adult male mice. Taken together, our results indicate that deletion of *Cntn6* is associated with the abnormal excitability of the AOS neural circuit and abnormal reproductive behavior in male mice.

## 2 MATERIALS AND METHODS

### 2.1 Animals

*Cntn6*‐deficient mice on a C57BL/6 background have been described previously (Takeda et al., 2003). Animals were housed in a temperature‐controlled facility and subjected to a 12‐h light/dark cycle with access to food and water ad libitum. All animal procedures were approved by the Capital Medical University Institutional Animal Care and Use Committee. Behavioral tests were performed in sexually naive male mice at 8–12 weeks of age, an hour after the onset of the dark cycle. Wild‐type males and estrous females (8–12‐week old), identified by a visual observation of the vaginal opening as described by Byers et al. (2012), were used in some behavioral tests. Each stimulus mouse was used only once.

### 2.2 Urine preference test

Urine preference test was conducted as described by Martel et al. (2009). Fresh urine from 3 to 4 sexually mature mouse donors was pooled according to gender immediately prior to the experiment. The same amount (50 μl) of male or estrus female urine was applied to the bottom of two clean glass slides (7.5 × 2.5 cm^2^), respectively. Slides were then fixed vertically by clips on the roof of the home cage (27 × 16 × 12 cm^3^). After being housed individually for 24 h, male mice were presented the urine slides for 3 min as shown in Figure 2a. A total of 15 *Cntn6^+/+^* and 15 *Cntn6^−/−^* male mice were tested. All tests were recorded on video, and the sniffing time, when the male mouse put its nose close to each slide, was measured by a blind observer.

### 2.3 Mate preference test

Evaluation of mate preference was performed in a Plexiglas three‐chamber box (60 × 30 × 22 cm^3^) as described by Hellier et al. (2018). Each chamber was 30 cm long and 20 cm wide, and the dividing walls had retractable doorways (8 × 5 cm) allowing access to each chamber. Each of the lateral chambers contained a cylinder (outer diameter: 7 cm, inner diameter: 6.4 cm, and height: 16 cm) formed by 18 evenly spaced metal rods (diameter: 0.3 cm) (Figure 2d). Male mice were acclimated to the three‐chamber box with two empty cylinders for 4 days prior to the test (10 min/day). On the day of the test, each male mouse was habituated to the three‐chamber box with two empty cylinders for 5 min before the experiment. Then, while keeping the male mouse in the middle chamber with the doors closed, a wild‐type male mouse was placed in one cylinder and a wild‐type estrous female mouse was placed in the other cylinder. The two doors were opened to allow the male mouse to explore all three chambers for 5 min. A total of 15 *Cntn6^+/+^* and 15 *Cntn6^−/−^* male mice were tested. During testing, male mouse activities were recorded on video and the time spent sniffing each cylinder was measured by a blind observer.

### 2.4 Mating behavior test

Mating behavior of male mice was assessed as described by Ferrero et al. (2013). Male mice were housed alone for 24 h prior to the tests. Mounting tests were performed in the home cage (27 × 16 × 12 cm^3^) with exposure to a wild‐type estrous female mouse. Mounting is defined as the male mouse riding on the back of the female mouse, whereas intromission is defined as the thrusting movements of the hindquarters. A total of 10 *Cntn6^+/+^* and 12 *Cntn6^−/−^* male mice were tested. The test was recorded for 30 min on video and mounting attempts were counted by a blind observer.

### 2.5 Buried food test

The buried food test was performed to assess the ability of the male mice to smell and find buried food as described by Canugovi et al. (2015). After 16 h of starvation, each male mouse was tested in an individual cage (27 × 16 × 12 cm^3^) containing 3‐cm‐deep clean bedding with one food pellet placed 1–1.5 cm below the bedding. The time taken to find the pellet was recorded; if the mouse did not find the pellet within 5 min, the test was ended and a time of 5 min was noted. A total of 10 *Cntn6^+/+^* and 12 *Cntn6^−/−^* male mice were tested. The analysis was done by a blind observer.

### 2.6 X‐gal staining

Three *Cntn6^+/−^* adult male mice (8–12‐week old) were transcardially perfused with ice‐cold phosphate‐buffered saline (PBS) containing 2% paraformaldehyde. The VNOs and the brains were removed and fixed overnight at 4°C. The brains were subsequently cryoprotected by sequential incubation overnight in PBS containing 15% and 30% sucrose. Cryosections (20 μm) were mounted on microscope slides, rinsed twice in PBS, and permeabilized in PBS containing 2 mM MgCl_2_ and 0.02% NP‐40 for 5 min. Sections were washed twice in PBS and incubated overnight at 37°C in X‐gal staining solution (5 mM K_3_Fe(CN)_6_, 5 mM K_4_Fe(CN)_6_·3H_2_O, 2 mM MgCl_2_, 20 mg/ml X‐gal). Sections were washed with PBS, counterstained with 1.2% neutral red for 30 s, rinsed in 75% ethanol, air dried, and cleared twice with xylene to visualize the brain architecture.

### 2.7 Immunostaining

Adult male mice (8–12‐week old, 3–5 mice for each genotype) were anesthetized and transcardially perfused with ice‐cold PBS containing 4% paraformaldehyde. Brains were dissected, fixed overnight at 4°C, and processed as described previously by Cho et al. (2012). Sections (20 μm) were harvested on microscope slides and incubated overnight at 4°C with the following primary antibodies: anti‐Gαo, 1:500 (MBL); anti‐olfactory marker protein (OMP), 1:500 (WAKO Chemicals); anti‐Npn2, 1:20 (R&D Systems); anti‐c‐Fos, 1:500 (Synaptic Systems); and anti‐VGlut2, 1:500 (Synaptic Systems). After rinsing in tris‐buffered saline, primary antibodies were detected with the appropriate Alexa‐488‐conjugated secondary antibody at 1:500 (Molecular Probes). Bandeiraea simplicifolia (BS) lectin, 1:1500 (Vector Laboratories) was applied with the secondary antibody.

### 2.8 c‐Fos staining measurement

Male mice were housed alone for 1 week. A total of five *Cntn6^+/+^* and five *Cntn6^−/−^* male mice were sacrificed following a 1‐h exposure to adult estrous female mice; three *Cntn6^+/+^* and three *Cntn6^−/−^* male mice with no exposure were used as controls. After fixation, brains were continuously cryosection at 25 μm thickness. Staining was performed as described in Section 2.7. The regions of interest were chosen based on the Paxinos Mouse Brain Atlas (Paxinos et al., 2019) and captured by confocal microscopy at the same magnification and fluorescent intensity (Leica, TSC SP8). The number of c‐Fos‐positive cells in the same area in one section from each mouse in these regions was counted manually using the ImageJ software. The location of each brain section relative to the bregma point is indicated in corresponding figures.

### 2.9 Electron microscopy

Adult male mice (12‐week old, four mice for each genotype) were transcardially perfused with PBS followed by fixative solution (2.5% glutaraldehyde in 0.1 M Na cacodylate buffer). AOBs were dissected to remove excess tissues and fixed overnight at 4°C. After repeated washing with 0.1 M Na cacodylate buffer, tissue samples were fixed in 2% osmic acid for 2 h and then stained with 4% aqueous uranyl acetate overnight at room temperature. Samples were dehydrated in an ethanol series, embedded with Spurr resin, and polymerized at 60°C for 24 h. Ultrathin sections (80 nm) were cut and micrographs were taken at 25,600× magnification (FEI, Tecnai G2 Spirit BioTWIN). The number of excitatory or inhibitory synapses in 20 sections from each mouse was counted manually.

### 2.10 Statistical analysis

Statistical analyses were performed using the GraphPad Prism 8 software. Parametric analysis was used when the data conformed to normal distribution; otherwise, nonparametric analysis was chosen. Mann–Whitney *U* test or unpaired *t* test were performed on urine preference test data. Welch's *t* test or unpaired *t* test were performed to analyze mate preference test data. Mann–Whitney *U* test was performed on the data regarding mounting behavior test and buried food test. Two‐way analysis of variance (ANOVA) with Bonferroni's multiple comparisons test was carried out on the number of c‐Fos‐positive cells. Unpaired *t* test was carried out on the number of excitatory and inhibitory synapses. Statistical significance for all tests was taken as *p* < .05; the *n* number for each analysis is stated in the figure legends.

## 3 RESULTS

### 3.1 Expression of *Cntn6* in the accessory olfactory system

**FIGURE 1:**
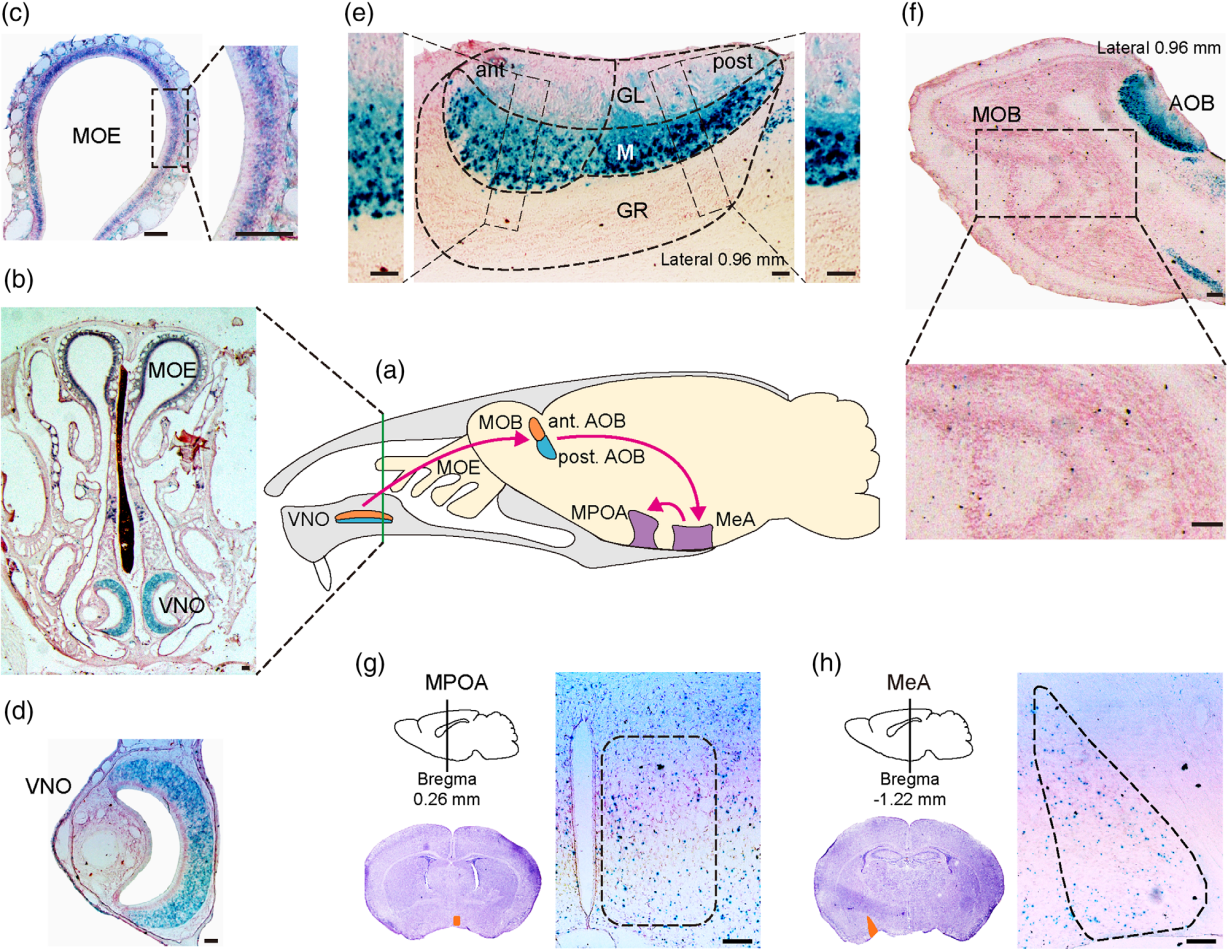
Expression patterns of *Cntn6* gene in the two olfactory systems monitored by *LacZ* expression in *Cntn6^+/−^* adult male mice. (a) Schematic representation of the two olfactory systems in the mouse brain. Pink arrows show the partial pathway of the accessory olfactory system. Green line shows the location of coronal section in (b). Expression of *Cntn6* is shown in coronal (b–d, g and h) and sagittal (e and f) sections of different regions. Black dashed rectangles in (c, e, and f) indicate higher magnification insets. Orange areas in (g and h) represent medial preoptic area (MPOA) and medial amygdala (MeA), respectively. Ant, anterior; AOB, accessory olfactory bulb; GL, glomerular layer; GR, granule cell layer; M, mitral cell layer; MOB, main olfactory bulb; MOE, main olfactory epithelium; post, posterior; VNO, vomeronasal organ. Scale bars, 0.1 mm

We first examined the expression pattern of *Cntn6* in the AOS and the MOS via X‐gal staining in *Cntn6^+/−^* adult male mice (Figure 1). *LacZ* signal was strong in the VNO and the AOB (Figure 1a,b,d,e), reflecting the expression of *Cntn6* in these regions, which is consistent with previous studies (Lee et al., 2000; Takeda et al., 2003). The MeA and the MPOA, which receive direct and/or indirect projections from mitral cells in the AOB, also exhibited sparse *LacZ* signal (Figure 1a,g,h). There was almost no *LacZ* signal in the MOB (Figure 1a,f), whereas *LacZ* signal was detected in the main olfactory epithelium (MOE) (Figure 1a–c). These results indicate that *Cntn6* is mainly expressed in the AOS.

### 3.2 *Cntn6*‐deficient male mice exhibit impaired mate preference

**FIGURE 2:**
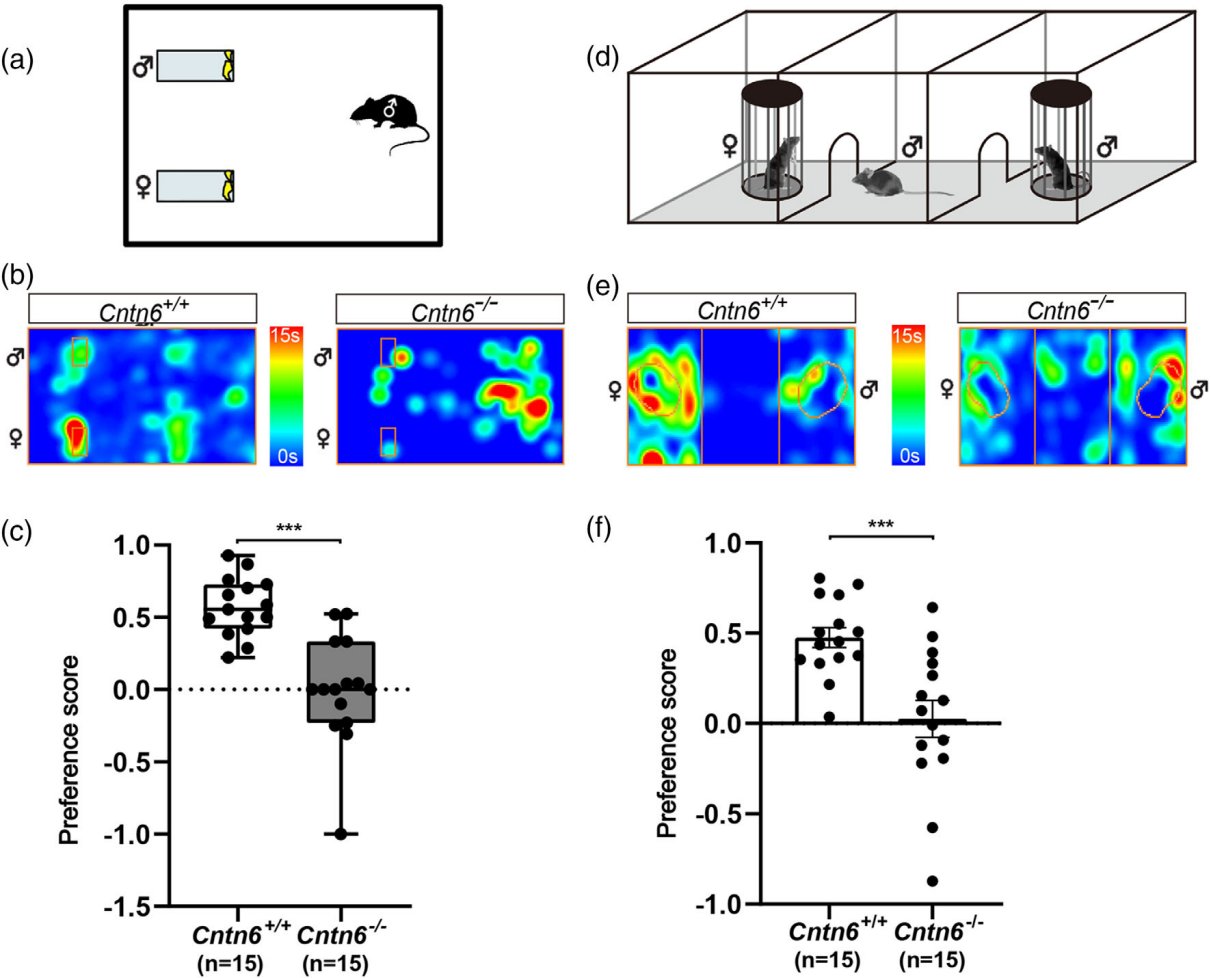
*Cntn6* ^−/−^ male mice show reduced mate preference toward estrous females. (a) Schematic illustration showing the urine preference test. (b) Representative heatmaps displaying the results of the urine preference test in (a). Each heatmap presents trajectory of an individual animal resulted from mouse tracking. The orange rectangles indicate the position of the slides coated with urine. (c) Quantification of the urine sniffing preference score in the urine preference test. Data did not conform to normal distribution by D'Agostino and Pearson test. Mann–Whitney *U* test, *p* < .0001 for *Cntn6^+/+^* versus *Cntn6^−/−^*. Data are presented as the median with interquartile range. Seven independent experiments. *n* = 15 for each genotype. (d) Schematic illustration showing the mate preference test. (e) Representative heatmaps displaying the results of the mate preference test in (d). Each heatmap presents trajectory of an individual animal resulted from mouse tracking. The orange circles indicate the position of the cylinders with mice placed. (f) Quantification of the sniffing time preference score in the mate preference test. *F* test to compare variances, *p* = .0265, Welch's *t* test, *p* = .0009 for *Cntn6^+/+^* versus *Cntn6^−/−^*. Seven independent experiments. *n* = 15 each genotype. Data are presented as the mean ± SEM. Individual data are presented as dots in (c) and (f). ***p < .001

To assess the function of CNTN6 in the AOS, the urine preference test was performed to evaluate whether male *Cntn6^−/−^* mice were able to distinguish between male and female mice (Figure 2a). Total sniffing time, time spent sniffing estrous female urine, and time spent sniffing male urine within a 3‐min window were collected and statistically analyzed. There was no statistical difference in total sniffing time between *Cntn6* ^+/+^ (27.3 ± 2.8 s, *n* = 15) and *Cntn6* ^−/−^ (20.2 ± 2.1 s, *n* = 15) male mice (*p* = .0534; Figure S1A). However, *Cntn6* ^+/+^ male mice spent more time sniffing female urine than male urine, whereas *Cntn6* ^−/−^ male mice spent the same period of time sniffing both female and male urine (Figure 2b). The preference score (time spent sniffing female mouse urine minus time spent sniffing male mouse urine as a proportion of total sniffing time) of *Cntn6* ^−/−^ male mice (−0.006 ± 0.097) are significantly lower than *Cntn6* ^+/+^ male mice (0.573 ± 0.052) (*p* < .0001; Figure 2c). The results indicate that *Cntn6* ^−/−^ male mice exhibit no significant preference for estrous female urine in comparison with their *Cntn6^+/+^* littermates.

Subsequently, we replaced urine with real mice that had full pheromonal cues (Figure 2d). Total sniffing time, time spent sniffing estrous female, and time spent sniffing intact male within a 5‐min window were collected and statistically analyzed. There was no statistical difference in total sniffing time between *Cntn6^+/+^* (113.1 ± 11.6 s, *n* = 15) and *Cntn6^−/−^* (115.9 ± 11.2 s, *n* = 15) male mice (*p* = .8603; Figure S1B). *Cntn6* ^+/+^ male mice spent more time sniffing estrous females rather than males; however, *Cntn6* ^−/−^ male mice spent roughly the same time on each side (Figure 2e). The preference score of *Cntn6^−/−^* male mice (0.026 ± 0.103) are significantly lower than *Cntn6^+/+^* male mice (0.476 ± 0.055) (*p* = .0009; Figure 2f). These results suggest that CNTN6 deficiency may reduce the preference of male mice to estrus females.

### 3.3 *Cntn6*‐deficient male mice exhibit reduced mounting behavior

**FIGURE 3:**
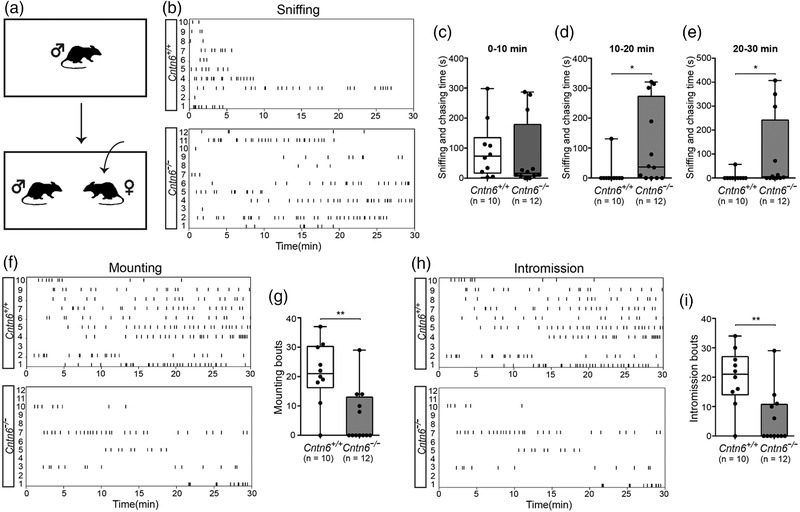
*Cntn6* ^−/−^ male mice show impaired reproductive behavior toward estrous females. (a) Schematic illustration showing mating behavior test. (b, f, h) Raster plots depict individual sniffing, mounting, intromission bouts of adult *Cntn6^+/+^*, and *Cntn6^−/−^* male mice toward adult estrous females during behavioral testing (30 min). Each tick indicates the onset of one sniffing, mounting, and intromission. (c–e) Quantitative analysis of sniffing and chasing time for every 10 min. *p* = .2902 for 0–10 min. *p* = .0124 for 10–20 min. *p* = .0248 for 10–30 min. (g and i) Quantitative analysis of mounting (*p* = .0023) and intromission bouts (*p* = .0026). Five independent experiments. *n* = 10 for *Cntn6^+/+^* and 12 for *Cntn6^−/−^* male mice, respectively. Data did not conform to normal distribution by D'Agostino and Pearson test. Mann–Whitney *U* test; Data are presented as the median with interquartile range. *p < .05, **p < .01

Pheromone‐triggered mate preference ultimately leads to the display of copulatory behavior. We next monitored the mating behavior of male mice exposed to estrous females (Figure 3a). As shown in Figure 3b,f, *Cntn6^+/+^* male mice sniffed estrous females until they started mounting and showed vigorous mounting attempts toward estrous females, whereas their *Cntn6^−/−^* littermates sniffed estrous females actively but with little to no mounting behavior. Interestingly, 7 out of 12 mice exhibited neither mounting nor intromission at all (Figure 3f,h). The difference of time spent in sniffing and chasing was not significant during the first 10 min (*p* = .2902; Figure 3c), but it was significant during the second (*p* = .0124; Figure 3d) and third 10 min (*p* = .0248; Figure 3e). Moreover, we found that the number of mounting bouts was significantly decreased in *Cntn6^−/−^* males as compared with *Cntn6^+/+^* males (*p* = .0026; Figure 3g). Intromission, as an indicator of the successful mounting, was also observed (Figure 3h). *Cntn6^−/−^* male mice displayed significantly less intromission than *Cntn6^+/+^* male mice (*p* = .0026; Figure 3i). These results suggest that CNTN6 deficiency may lead to reduced mating behavior in male mice.

**FIGURE 4:**
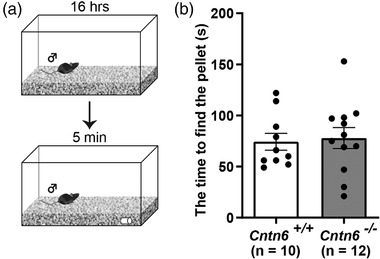
Normal performance of *Cntn6* ^−/−^ male mice in buried food test. (a) Schematic illustration showing the buried food test. (b) Quantification of time spent in finding buried food pellet (*p* = .7921). Five independent experiments. *n* = 10 for *Cntn6^+/+^* and 12 for *Cntn6^−/−^* male mice, respectively. Data are presented as the mean ± SEM; unpaired *t* test

Although the MOS mainly processes volatile odors, previous studies have indicated that information processing by the MOS is also necessary for the initiation of mating behavior (Choi et al., 2016). We then examined the function of the MOS using the buried food test (Figure 4a). *Cntn6^+/+^* and *Cntn6^−/−^* male mice took a similar length of time to find the buried food pellets, with no significant difference in the average latency (*p* = .7921; Figure 4b), which suggest that the MOS may function normally in *Cntn6^−/−^* male mice, consistent with the low expression of *Cntn6* in the MOS (Figure 1).

### 3.4 CNTN6 deficiency does not affect the gross structure of the AOS

**FIGURE 5:**
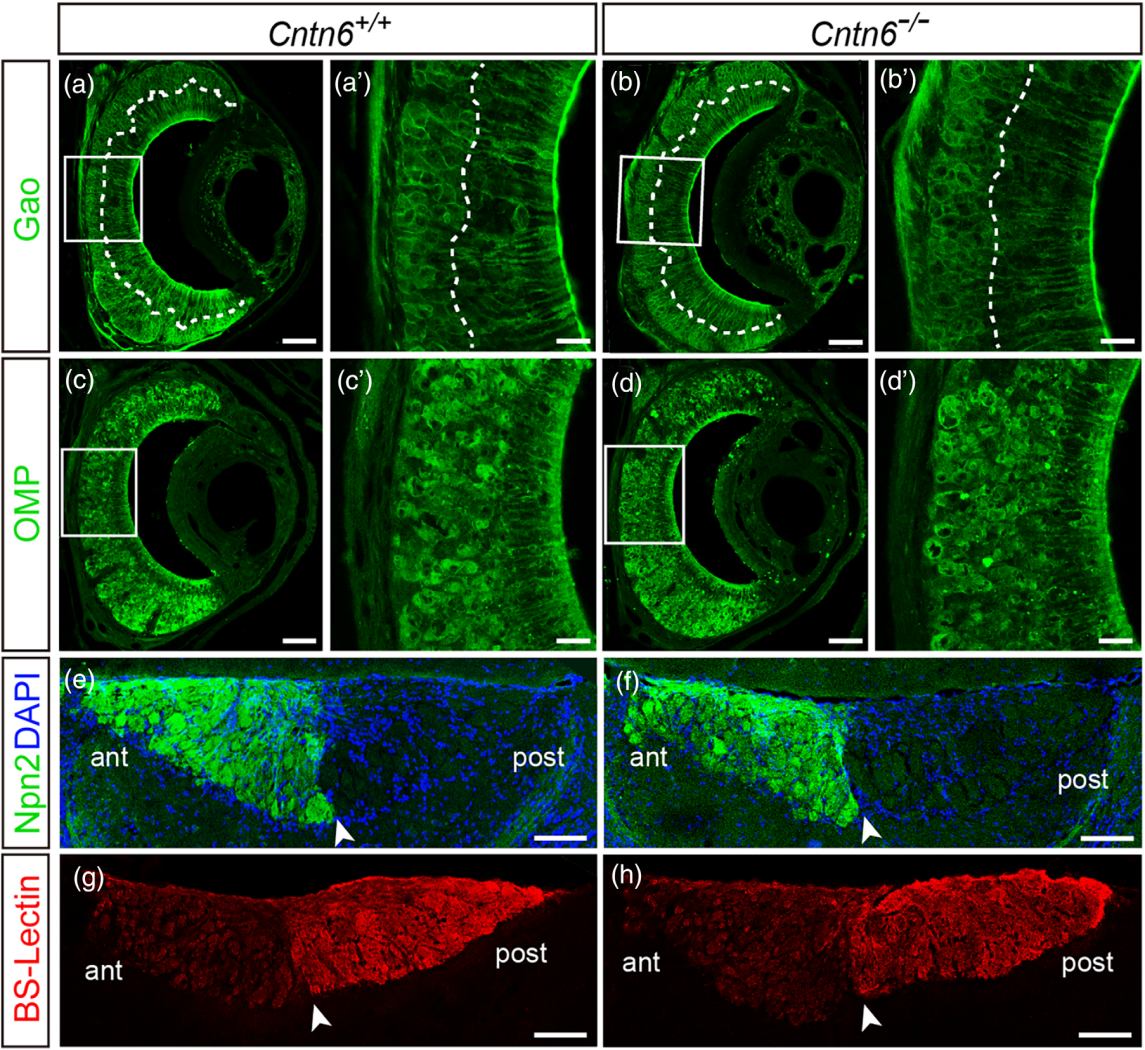
The gross structure of the vomeronasal organ (VNO) and accessory olfactory bulb (AOB) is similar between *Cntn6^+/+^* and *Cntn6^−/−^* male mice. (a–d) Coronal sections of the VNO from *Cntn6^+/+^* and *Cntn6^−/−^* male mice stained with antibodies against Gαo and olfactory marker protein (OMP), respectively. The dotted line separates the apical and basal layers of the VNO. (a′–d′) Higher magnification of the respective insets in (a–d). (e–h) Sagittal sections of the AOB from *Cntn6^+/+^* and *Cntn6^−/−^* male mice stained with anti‐Npn2 antibody and Bandeiraea simplicifolia (BS)‐lectin, respectively. Arrowheads denote the border between the anterior (ant) and posterior (post) regions of the AOB. Representative figures from three independent experiments (*n* = 3 mice for each genotype). Scale bars, 50 μm (a′–d′), 100 μm (a–d); 250 μm (e–h)

The altered behavior observed in *Cntn6^−/−^* male mice suggested that either development or function of the AOS was affected by deletion of *Cntn6*; thus, we first examined whether the segregation and maturation of the VSNs were affected. Immunostaining of VNO sections using an anti‐Gαo antibody revealed that Gαo‐expressing VSNs were located at the basal region of the VNO in both *Cntn6^+/+^* and *Cntn6^−/−^* male mice, indicating that the segregation of basal and apical VSNs is intact in *Cntn6^−/−^* mice (Figure 5a,a′,b,b′). As shown in Figure 5c,c′,d,d′, there was no difference in the expression of OMP, a marker for VSN maturation, between *Cntn6^+/+^* and *Cntn6^−/−^* male mice. To determine whether the dichotomy of VSN axons to the AOB was affected, we examined the projection of apical VSN axons to the anterior regions of the AOB. Sections of the AOB from *Cntn6^+/+^* and *Cntn6^−/−^* mice were stained with an antibody against Npn2, to visualize axonal projection from the apical VSNs, and with BS lectin, to identify the segregation of anterior and posterior regions of the AOB. Npn2‐expressing axons projected exclusively to the anterior half of the AOB in both *Cntn6^+/+^* and *Cntn6^−/−^* male mice (Figure 5e,f). Similarly, the border of the anterior and posterior regions of the AOB was clear in both *Cntn6^+/+^* and *Cntn6^−/−^* male mice as revealed in the BS‐lectin staining (Figure 5g,h). Taken together, these results indicate that CNTN6 does not influence the formation of VSNs or their axonal projections.

### 3.5 Loss of CNTN6 disturbs the information processing pathway of the AOS

**FIGURE 6:**
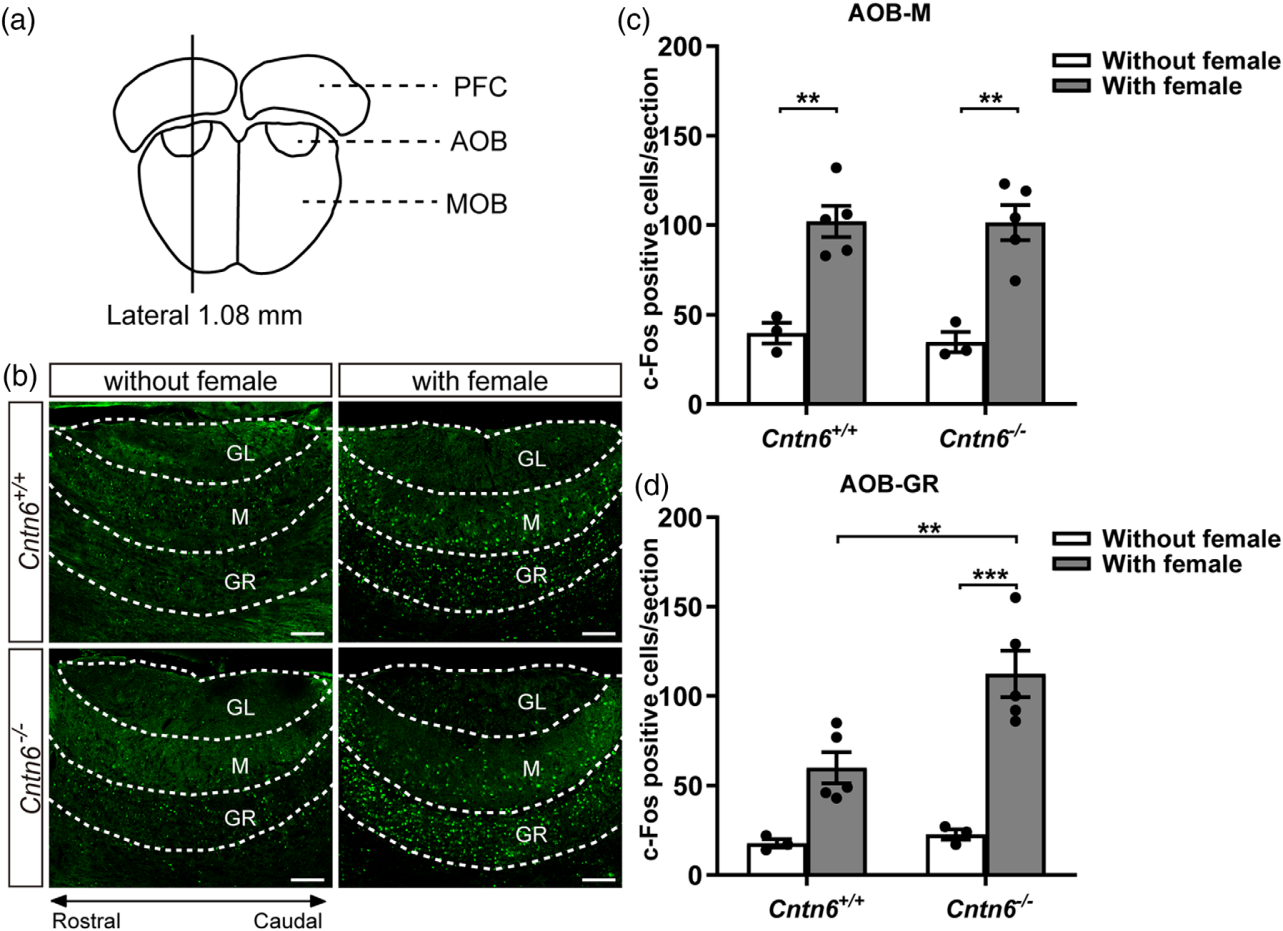
Contactin‐6 (CNTN6) deficiency affects activity of granule cells rather than mitral cells in the accessory olfactory bulb (AOB) upon female exposure. (a) Schematic illustration showing the location of the AOB sections. (b) Immunostaining using an anti‐c‐Fos antibody in sagittal sections of the AOB from *Cntn6^+/+^* and *Cntn6^−/−^* male mice with or without a 1‐h exposure to estrous females. (c and d) Quantitation of c‐Fos‐positive cells in one section containing the AOB of each mouse. For the mitral cell layer (c), *p* = .0028 for *Cntn6^+/+^* (without vs. with female exposure); *p* = .0016 for *Cntn6^−/−^* (without vs. with female exposure). For the granule cell layer (d), *p* = .0073 for those with female exposure (*Cntn6^+/+^* vs. *Cntn6^−/−^*); *p* = .0003 for *Cntn6^−/−^* (without vs. with female). Three independent experiments. Without female exposure, *n* = 3 mice for each genotype. With female exposure, *n* = 5 mice for each genotype. Data are presented as the mean ± SEM; two‐way analysis of variance (ANOVA) with Bonferroni's post hoc test. GL, glomerular layer; GR, granule cell layer; M, mitral cell layer; MOB, main olfactory bulb; PFC, prefrontal cortex. Scale bars, 250 μm (b). **p < .01, ***p < .001

**FIGURE 7:**
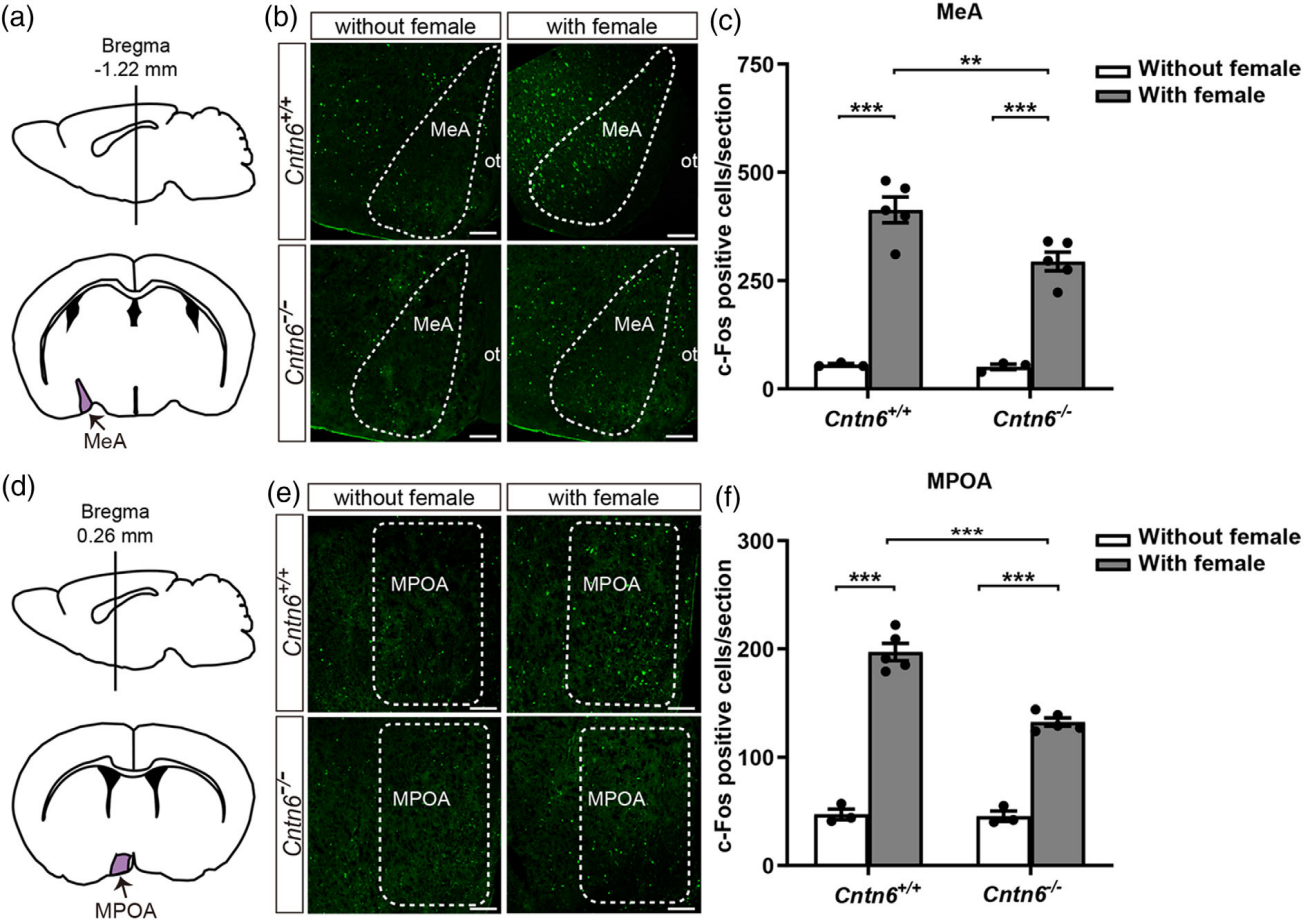
Neural activity upon female exposure was decreased in both the medial amygdala (MeA) and the medial preoptic area (MPOA) in *Cntn6^−/−^* male mice. Schematic illustration showing the location of the MeA (a) and the MPOA (d) sections. Immunostaining using an anti‐c‐Fos antibody in coronal sections of the MeA (b) and the MPOA (e) from *Cntn6^+/+^* and *Cntn6^−/−^* male mice with or without a 1‐h exposure to estrous females. Quantitation of c‐Fos‐positive cells in one section containing the MeA (c) or the MPOA (f) from each mouse. In the MeA (c), *p* = .0091 for those with female exposure (*Cntn6^+/+^* vs. *Cntn6^−/−^*); *p* < .0001 for *Cntn6^+/+^* (without vs. with female exposure); *p* < .0001 for *Cntn6^−/−^* (without vs. with female exposure). In the MPOA (F), *p* < .0001 for those with female exposure (*Cntn6^+/+^* vs. *Cntn6^−/−^*); *p* < .0001 for *Cntn6^+/+^* (without vs. with female exposure); *p* < .0001 for *Cntn6^−/−^* (without vs. with female exposure). Three independent experiments. Without female exposure, *n* = 3 mice for each genotype. With female exposure, *n* = 5 mice for each genotype. Data are presented as the mean ± SEM; two‐way analysis of variance (ANOVA) with Bonferroni's post hoc test. ot, optic tract. Scale bars, 250 μm (b and e). **p < .01, ***p < .001

As the gross structure of the AOS appeared normal in *Cntn6* ^−/−^ mice, we next evaluated whether the neural circuit of the AOS was functioning correctly by determining the expression of the immediate early gene *c‐Fos* as a marker of cell activity. We assessed in vivo c‐Fos immunostaining in the AOB (Figure 6a,b) and regions receiving AOB projection implicated in reproductive behaviors, the MeA and the MPOA (Figure 7a,d), with and without female exposure. A two‐way ANOVA was carried out to examine the effects of two independent variables (genotype, stimulus) and their interaction on the number of activated neurons in the AOB. The results reveal a significant effect of stimulus [*F*(1, 12) = 48.64, *p* < .0001] but not genotype [*F*(1, 12) = 0.09158, *p* = .7674] and their interaction [*F*(1, 12) = 0.05653, *p* = .8161] on the number of activated mitral cells; significant effects of genotype [*F*(1, 12) = 7.469, *p* = .0182], stimulus [*F*(1, 12) = 39.54, *p* < .0001], and their interaction [*F*(1, 12) = 5.093, *p* = .0435] on the number of activated granule cells. After female mice exposure, post hoc analysis showed that there was no significant difference in the number of activated mitral cells between *Cntn6^+/+^* and *Cntn6^−/−^* male mice (*p* > .9999; Figure 6c), whereas *Cntn6^−/−^* male mice displayed a significantly increased activated granule cells compared to their *Cntn6^+/+^* littermates (*p* = .0073; Figure 6d).

In the MeA and the MPOA, the number of activated neurons was also increased in both in *Cntn6^+/+^* male mice and *Cntn6^−/−^* male mice with female exposure (Figure 7b,e). As revealed by two‐way ANOVA, there were significant effects of genotype [*F*(1, 12) = 6.299, *p* = .0274], stimulus [*F*(1, 12) = 149.0, *p* < .0001] and their interaction [*F*(1, 12) = 5.382, *p* = .0388] on the number of activated neurons in the MeA; significant effects of genotype [*F*(1, 12) = 26.86, *p* = .0002], stimulus [*F*(1, 12) = 343.0, *p* < .0001], and their interaction [*F*(1, 12) = 24.22, *p* = .0004] on the number of activated neurons in the MPOA. The increased number of activated neurons in *Cntn6^−/−^* male mice was significantly decreased compared with their *Cntn6^+/+^* littermates both in the MeA (*p* = .0091; Figure 7c) and the MPOA (*p* < .0001; Figure 7f) as revealed by post hoc tests. These results suggest that CNTN6 deficiency affects the activity of AOS circuitry, including the AOB, the MeA, and the MPOA.

Increased activation of granule cells in the AOB of *Cntn6^−/−^* male mice upon female exposure suggests abnormal synaptic transmission in the AOB. Therefore, we examined the excitatory synapses between mitral and granule cells (located in the mitral cell layer of the AOB) using electron microscopy (Figure S2A). Excitatory synapses have a postsynaptic density (PSD) with a thickness of 20–50 nm. In contrast, the PSD in inhibitory synapses contains a ∼12 nm structure from the postsynaptic membrane. *Cntn6^−/−^* male mice exhibited a significantly increased number of excitatory synapses as compared with *Cntn6^+/+^* male mice, whereas there was no significant difference in the number of inhibitory synapses (Figure S2B,C). These results suggest that interfered AOS circuitry in CNTN6 deficiency male mouse is probably resulted from affected excitatory synapses between mitral and granule cells in AOB.

## 4 DISCUSSION

CNTN6, as a neuronal IgCAM, has been implicated in the development and function of the neural system (Huang et al., 2011, 2012; Lee et al., 2000; Mu et al., 2018; Sakurai et al., 2009; Takeda et al., 2003; Ye et al., 2008). Here, we report that *Cntn6* is highly expressed in the AOS as compared with the MOS in mice and is involved in the proper functioning of the AOS. We demonstrate that the loss of CNTN6 leads to impairment in mate preference and behavior in adult male mice. Interestingly, CNTN6 dysfunction does not affect VSN development and the projection of their axons to AOB or the gross architecture of the VNO and the AOB. However, compared with *Cntn6^+/+^* adult male mice, *Cntn6^−/−^* adult male mice shows an increased number of activated granule cells in AOB and decreased neural activity in the MeA and the MPOA after female exposure. Moreover, *Cntn6^−/−^* adult male mice also possess more excitatory synapses between mitral and granule cells in AOB. These results suggest that deletion of *Cntn6* leads to abnormal male mice mating behavior probably via affecting the neural activity of AOS circuitry rather than interfering with the AOS structure.

The AOS is generally considered to be involved in the detection of pheromones that affect reproductive behavior through its close connections with the reproductive hypothalamus (Keller et al., 2009; Keverne, 2004; Rodriguez, 2004). The MOS is believed to be a general analyzer that detects complex odors present in the environment of individuals (Firestein, 2001; Keller et al., 2009). To date, the functional dichotomy between the AOS and MOS remains unclear; thus, the *Cntn6* expression pattern in both systems was analyzed. We found that *Cntn6* is expressed widely and highly in the VNO, with no specificity for apical or basal VSNs, indicating that it may be involved in almost all the information processes of the VNO. Moreover, there is a robust expression of *Cntn6* in the mitral cell layer of the AOB and sparse expression in the MeA and the MPOA. In contrast, although a diffuse expression pattern of *Cntn6* was observed in the MOE, barely any expression was detected in the MOB, suggesting that *Cntn6* is mainly expressed in the AOS and that CNTN6 deficiency may impair AOS function.

Considering the guiding function of the AOS in sex‐specific social and reproductive behavior, we therefore performed behavior tests in male mice, to evaluate the effect of CNTN6 deficiency on the AOS.

In comparison with *Cntn6^+/+^* adult male mice, the mate preference related to both urine odors and direct contact with estrous females versus intact males was significantly reduced in *Cntn6^−/−^* adult male mice (Figure 2). Subsequently, we tested reproductive behavior caused by pheromone‐triggered mate preference. Consistently, a number of sniffing, mounting attempts, and intromission bouts were also significantly reduced in *Cntn6^−/−^* males as compared with those in *Cntn6^+/+^* males and 7 out of 12 *Cntn6^−/−^* males did not mount or copulate at all (Figure 3). We also performed buried food test to assess the MOS function of CNTN6 deficiency male mice. Our results suggest that general odor detection, at least for food, is not affected in *Cntn6^−/−^* male mice (Figure 4), although the role of MOS in the change of mating behavior observed in *Cntn6^−/−^* males cannot be fully excluded at this point. Considering that *Cntn6* is expressed in the MOE, more thoroughly designed tests are necessary to determine if CNTN6 affects the MOS function.

The question remained regarding what is responsible for changes in mating preference in *Cntn6^−/−^* male mice; therefore, we assessed the development of the AOS, including VNO and AOB dichotomy, VSN projections, and AOB glomeruli formation. Interestingly, there were no obvious changes in the gross architecture in *Cntn6^−/−^* male mice (Figure 5), suggesting that CNTN6 deficiency may affect the AOS function in a different manner. It is not clear whether CNTN6 disturbs certain types of the VRs in the VNO and subsequent information processing, which can affect those reproductive behaviors. A recent study revealed that members of V1re clade recognizes gender‐identifying cues in female urine, whereas members of the V1rj clade detect urinary estrus signals and sulfated estrogen compounds (Haga‐Yamanaka et al., 2014). The pheromones used in this paper were complex mixture. Identifying the pheromones would be the first step for the further research.

Huang et al. (2019) reported that CNTN6 deficiency promoted synapse reformation between serotonergic raphespinal tract regenerative axons and motor neurons. Also, *CNTN6* has been regarded as a candidate risk gene for neurodevelopmental and neuropsychiatric disorders (Hu et al., 2015; Huang et al., 2017; Kashevarova et al., 2014; Kerner et al., 2011; Oguro‐Ando et al., 2017; Pinto et al., 2010), suggesting an important role of CNTN6 in neural development, especially excitatory–inhibitory balance. In the AOB, there are reciprocal dendrodendritic synapses between mitral and granule cells that regulate excitatory–inhibitory balance. Glutamate released from mitral cell dendrites activates granule cells, which in turn mediate GABAergic dendrodendritic inhibition of mitral cell dendrites (Jia et al., 1999), forming an inhibitory feedback to regulate mitral cell activity (Kaba & Keverne, 1988; Li et al., 1990).

We found that c‐Fos activation upon female exposure was similar in the mitral cells of the AOB, but was increased in granule cells, and was decreased in the MeA and the MPOA (Figures 6 and 7) in *Cntn6^−/−^* adult males as compared with their *Cntn6^+/+^* counterparts. Lower activation of the MeA and the MPOA in *Cntn6^−/−^* males may contribute to the behavioral changes; nevertheless, the increased number of activated granule cells with no obvious changes in mitral cells suggests that the dendrodendritic synapses between them may be disturbed. Indeed, electron microscopy showed an increased number of excitatory synapses in *Cntn6^−/−^* males (Figure S2), suggesting that interference in the reciprocal dendrodendritic synapses and enhanced inhibitory feedback between mitral and granule cells may be one of the mechanisms underlying AOS dysfunction in *Cntn6^−/−^* male mice. Previous studies revealed that NMDA receptors, mGluR1 and mGluR2, influence dendrodendritic inhibition between mitral cells and granule cells (Castro et al., 2007; Jia et al., 1999; Smith et al., 2009; Taniguchi et al., 2001, 2013). Our work supports the important role of dendrodendritic inhibition in the AOS neural circuit and provides the possibility that CNTN6 involves in synaptic mechanism underlying reproductive behavior. However, it remains to be examined whether CNTN6 deficiency also directly disturbs the MeA, the MPOA, and other downstream areas involved in the reproductive behaviors, such as BNST and MeApd. The precise molecular and functional mechanisms require further investigation.

Recent reports reveal *CNTN6* as a candidate susceptibility gene of autism spectrum disorder (ASD) (Doan et al., 2016; Hu et al., 2015; Iossifov et al., 2014; Kashevarova et al., 2014; Mercati et al., 2017; Poot, 2014; Schmitz‐Abe et al., 2020; van Daalen et al., 2011), a spectrum disorder in which social impairment and repetitive behavior are the core symptoms. However, the precise mechanism of *CNTN6*’s involvement in the pathogenesis of ASD is poorly understood. Here, we observed an impaired social–sexual behavior of *Cntn6*‐deficient male mouse, which may result from the abnormal excitability of the AOS neural circuit. Our result provides a possible research direction to investigate the involvement of *CNTN6* in the pathogenesis of ASD.

## 5 CONCLUSION

*Cntn6* is highly expressed in the AOS, and *Cntn6*‐deficient male mice exhibit abnormal mate preference and reproductive behavior related to AOS. CNTN6 deficiency barely affects the gross structure of the AOS. However, decreased cell activity in the MeA and the MPOA was observed, which may be attributed to more activated granule cells and increased excitatory synapses between mitral cells and granule cells in the AOB. Thus, our results show that the impaired reproductive behavior of *Cntn6*‐deficient male mice may result from abnormal excitability of the AOS neural circuit, rather than structural changes.

## CONFLICT OF INTEREST

The authors declare that there is no conflict of interest that could be perceived as prejudicing the impartiality of the research reported.

## FUNDING INFORMATION

National Natural Science Foundation of China (Grant Numbers: 31971116, 31801208, 91854209); R&D Program of Beijing Municipal Education Commission (Grant Number: KM201910025025); and Beijing Municipal Natural Science Foundation (Grant Number: 5202005)

### PEER REVIEW

The peer review history for this article is available at https://publons.com/publon/10.1002/brb3.2893.

## Supporting information


**Figure S1** Total time spent on sniffing the stimuli. (A) Total sniffing time in the urine preference test. Unpaired *t* test, *p* = 0.0534. (B) Total sniffing time in the mate preference test. Unpaired *t* test, *p* = .8603. Data are presented as the mean ± SEM. Individual data are presented as dots in (A) and (B). *n* = 15 each genotype. No statistical significance was detected for both (A) and (B).

**Figure S2** *Cntn6^−/−^* male mice possess a higher number of excitatory synapses between mitral and granule cells as compared with *Cntn6^+/+^* male mice. (A) Schematic illustration showing the location of the AOB sections. The regions quantified were located in the mitral cell layer. (B) Electron micrographs of the mitral cell layer of the AOB in *Cntn6^+/+^* and *Cntn6^−/−^* male mice. Arrowheads indicate excitatory synapses. Arrows indicate inhibitory synapses. (C) Quantification of excitatory (*p* = .0007) and inhibitory synapses (*p* = .6525) in electron micrographs of the mitral cell layer of *Cntn6^+/+^* and *Cntn6^−/−^* male mice. Sum of 20 sections for each mouse, 4 mice for each genotype. Data are presented as the mean ± SEM; unpaired *t* test. AOB, accessory olfactory bulb; GR, granule cell layer; M, mitral cell layer; MOB, main olfactory bulb; PFC, prefrontal cortex. Scale bars, 2 μm (F). ***p < .001.

## Data Availability

The data that support the findings of this study are available from corresponding author upon reasonable request.
