# Supplementary figures and images for: Contactin‐6‐deficient male mice exhibit the abnormal function of the accessory olfactory system and impaired reproductive behavior
Source: Brain Behav. 2023 Mar 1;13(4):e2893. doi: 10.1002/brb3.2893 (PMC10097056; doi:10.1002/brb3.2893)

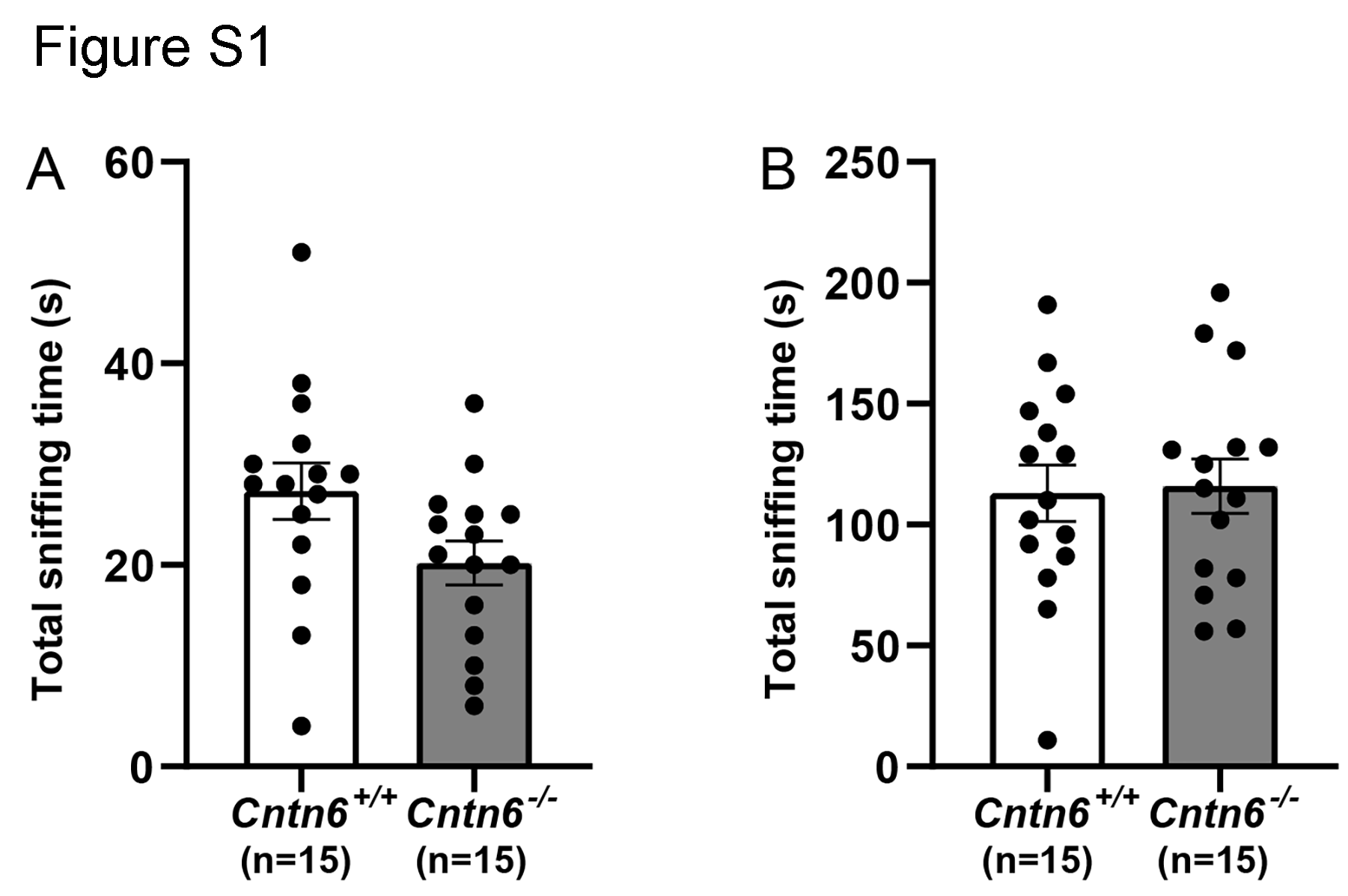

Supplement: Supplementary file 1 — Figure S1 Total time spent on sniffing the stimuli. (A) Total sniffing time in the urine preference test. Unpaired t test, p = 0.0534. (B) Total sniffing time in the mate preference test. Unpaired t test, p = .8603. Data are presented as the mean ± SEM. Individual data are presented as dots in (A) and (B). n = 15 each genotype. No statistical significance was detected for both (A) and (B). [file BRB3-13-e2893-s001.tif]

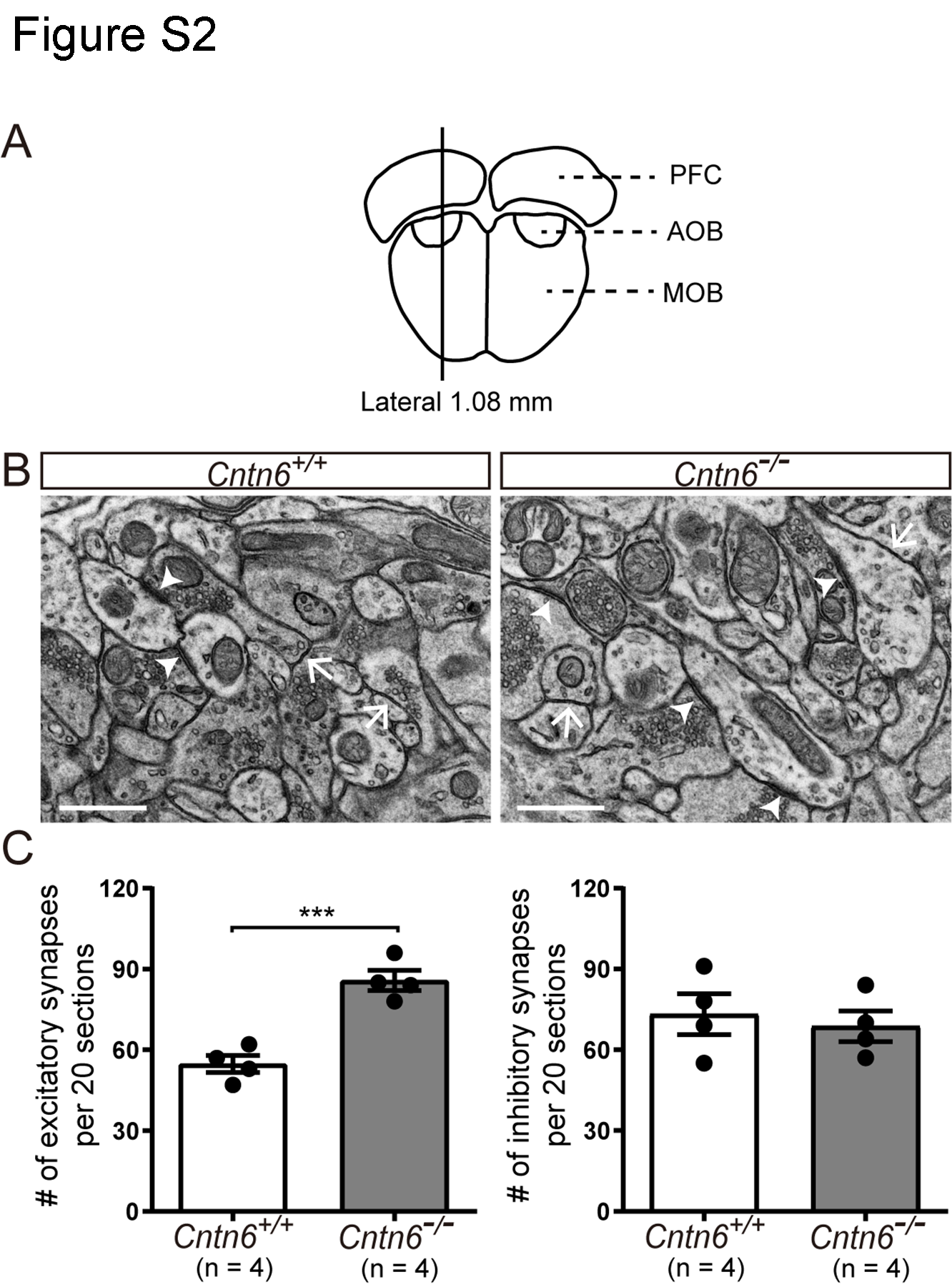

Supplement: Supplementary file 2 — Figure S2 Cntn6−/− male mice possess a higher number of excitatory synapses between mitral and granule cells as compared with Cntn6+/+ male mice. (A) Schematic illustration showing the location of the AOB sections. The regions quantified were located in the mitral cell layer. (B) Electron micrographs of the mitral cell layer of the AOB in Cntn6+/+ and Cntn6−/− male mice. Arrowheads indicate excitatory synapses. Arrows indicate inhibitory synapses. (C) Quantification of excitatory (p = .0007) and inhibitory synapses (p = .6525) in electron micrographs of the mitral cell layer of Cntn6+/+ and Cntn6−/− male mice. Sum of 20 sections for each mouse, 4 mice for each genotype. Data are presented as the mean ± SEM; unpaired t test. AOB, accessory olfactory bulb; GR, granule cell layer; M, mitral cell layer; MOB, main olfactory bulb; PFC, prefrontal cortex. Scale bars, 2 μm (F). ***p < .001. [file BRB3-13-e2893-s002.tif]
